# Multiparametric analysis of the effectiveness of cisplatin on cutaneous squamous carcinoma cells using two different types of adjuvants

**DOI:** 10.1371/journal.pone.0230022

**Published:** 2020-03-06

**Authors:** Silvia Gil, Eduardo Solano, Francesc Martínez-Trucharte, Jordi Martínez-Esaín, Ana J. Pérez-Berná, José Javier Conesa, Christina Kamma-Lorger, Mercè Alsina, Manel Sabés

**Affiliations:** 1 Hospital Clínic de Barcelona, Barcelona, Spain; 2 Hospital Parc Taulí, Sabadell, Barcelona, Spain; 3 ALBA Synchrotron Light Source, Barcelona, Spain; 4 Departament de Química, Universitat Autònoma de Barcelona, Bellaterra, Spain; 5 Australian Synchrotron–Australian Nuclear Science and Technology Organisation, Clayton, Victoria, Australia; 6 Unitat de Biofísica, Faculty of Medicine, Universitat Autònoma de Barcelona, Bellaterra, Barcelona, Spain; VIT University, INDIA

## Abstract

The objective of this study was to regulate the cytotoxicity of cisplatin (cisPt) minimizing its adverse effects. For this purpose, the lowest cisPt concentration needed to obtain a significant positive response in cutaneous squamous cell carcinoma (cSCC) was explored. Two adjuvant agents as gold nanoparticles (AuNP) and chelating tricine were tested as enhancers in cisPt treatment. Effectiveness of all treatments was assessed by means of biochemical techniques, which offer quantitative data, as well as two microscopy–based techniques that provided qualitative cell imaging. The present work confirms the effectiveness of free cisplatin at very low concentrations. In order to enhance its effectiveness while the side effects were probably diminished, cisPt 3.5 μM was administered with AuNP 2.5 mM, showing an effectiveness practically equal to that observed with free cisPt. However, the second treatment investigated, based on cisPt 3.5 μM combined with tricine 50 mM, enhanced drug effectiveness, increasing the percentage of cells dying by apoptosis. This treatment was even better in terms of cell damage than free cisPt at 15 μM. Images obtained by TEM and cryo-SXT confirmed these results, since a notable number of apoptotic bodies were detected when cisPt was combined with tricine. Thus, tricine was clearly a better adjuvant for cisPt treatments.

## Introduction

*Cis*-diamminedichloroplatinum (II) (cisplatin or cisPt) is an alkylating neutral complex, widely used as a chemotherapeutic agent against a broad range of cancers, showing substantial therapeutic impact against most carcinoma-like tumors [1,2]. Within the cell, cisPt is known to cause a distortion of the structure of the DNA double helix [3,4] affecting DNA replication, and inhibiting the major nuclear repair pathway of cisPt-DNA adducts [3], which induces apoptotic death [2]. Therefore, cisPt is considered a highly toxic agent, which can be used for chemotherapeutic treatments.

Unfortunately, cisPt administration results in severe systemic clinical toxicity, which includes nausea, nephrotoxicity, gastrointestinal toxicity, peripheral neuropathy, asthenia, and ototoxicity [5]. As a consequence, the clinical dose that can be used is a key limitation [6].

A previous study reported higher effectiveness of cisPt as a chemotherapy agent against cutaneous squamous cell carcinoma (cSCC) in comparison with the commonly used 5-fluorouracil [7]. Results showed that lower cisPt concentrations and increased treatment time were required to achieve higher effectiveness.

Therefore, it was crucial to determine the minimum cisPt concentration (in the order of μM) that was required to obtain a significant decrease in cSCC cell survival, without causing serious side effects. This minimum concentration could be further compensated by using adjuvant agents which convey the effective cisPt dose to the cell nucleus by facilitating passage through the cell membrane. In the present study, two different types of adjuvants were investigated based on two different action principles for increasing cisPt efficacy against cSCC: gold nanoparticles (AuNP) as carriers, and tricine as a chelating agent:

1. AuNP: Gold nanoparticles have been shown to enter the cell easily via endocytosis [8]. The coordination of AuNP with cisPt was achieved via a pH-sensitive bond based on thiol-stabilized particles with carboxyl functionality ligands [8]. In particular, the thiolate acid used in this work is 11-mercaptoundecanoic acid (–MUA), which binds to AuNP surfaces through SH-Au bonds. Hence, carboxylic groups allowed cisPt binding, enhancing its entry into the cell by passive diffusion. Inside the cell, the acidic pH of vesicles as endosomes could break the bond between carboxylic acid and cisPt, facilitating the diffusion of free cisPt until the nucleus. AuNP also may act as radiation enhancers for any subsequent radiation treatment.
2. Tricine: N-[tris(hydroxymethyl)methyl]glycine, or tricine, is widely used as a biological buffer for a pH range of 7.4 to 8.8. It is well known that this molecule can act as a chelator (i.e. forming cyclic complexes by coordination of its polydentate ligands with some metals such as copper and zinc [9]). The decrease in the presence of Cu^2+^ inside cells caused by tricine could enhance cisPt effectiveness, since the activated form of the drug (formed by Pt^2+^) might also enter the cell through the copper transporter proteins located on the plasmatic membrane.

To further investigate and assess the best strategy to obtain maximum benefit of cisPt administration, common laboratory methodologies (i.e. quantification of living and dead cells, and the measurement of the metabolic activity of surviving cells) were applied in the current study. Moreover, advanced characterization tools to evaluate the penetration and distribution of AuNP inside the cells were needed, since the AuNP adjuvant effect would depend on the extent of penetration and the location of these nanoparticles. For that, conventional Transmission Electron Microscopy (TEM) and cryo-synchrotron soft X-ray tomography (Cryo-SXT) were employed in order to study the effect of cisPt treatment with both adjuvants on different cell organelles. These complementary techniques covered an imaging resolution range from nanometers to microns. Cryo-SXT is the only image-based technique that allows 3D investigation on a nanometric scale and only requires sample vitrification to perform the 3D volume investigation. [10]

These techniques revealed that tricine was a more effective adjuvant for cisPt treatment than AuNP, although the underlying mechanism is not yet understood.

## Methods

### Cell culture and cisPt treatments

A431NS human squamous cutaneous carcinoma cell line was obtained from the American Type Culture Collection. Cells were cultured in flasks of 25 cm^2^ with Dulbecco’s Modified Eagle’s Medium (DMEM) supplemented with 1% penicillin/streptomycin, and with 10% fetal calf serum to reach confluence. Two hundred thousand cells were seeded per well in 24-well plates, and incubated for 24 h at 37°C and 5% CO_2_. Subsequently, all supernatants were removed and replaced by either medium or CisPt (*Ferrer Farma*, 1 mg ml^-1^) diluted in medium to reach a final concentration of 3.5, 7 or 15 μM, and the cells were incubated for a further 24 h. Finally, cisPt 3.5 μM was determined to be the working concentration after testing all three, and it was added free, combined with AuNP 2.5 mM or mixed with tricine 50 mM (pH = 7, Bioworld).

This AuNP concentration was chosen considering already published data elsewhere [11]. For tricine, a millimolar scale concentration was chosen as described in the literature [9,12,13].

### Gold nanoparticles and ligand exchange between MUA-citrate

**Fig 1:**
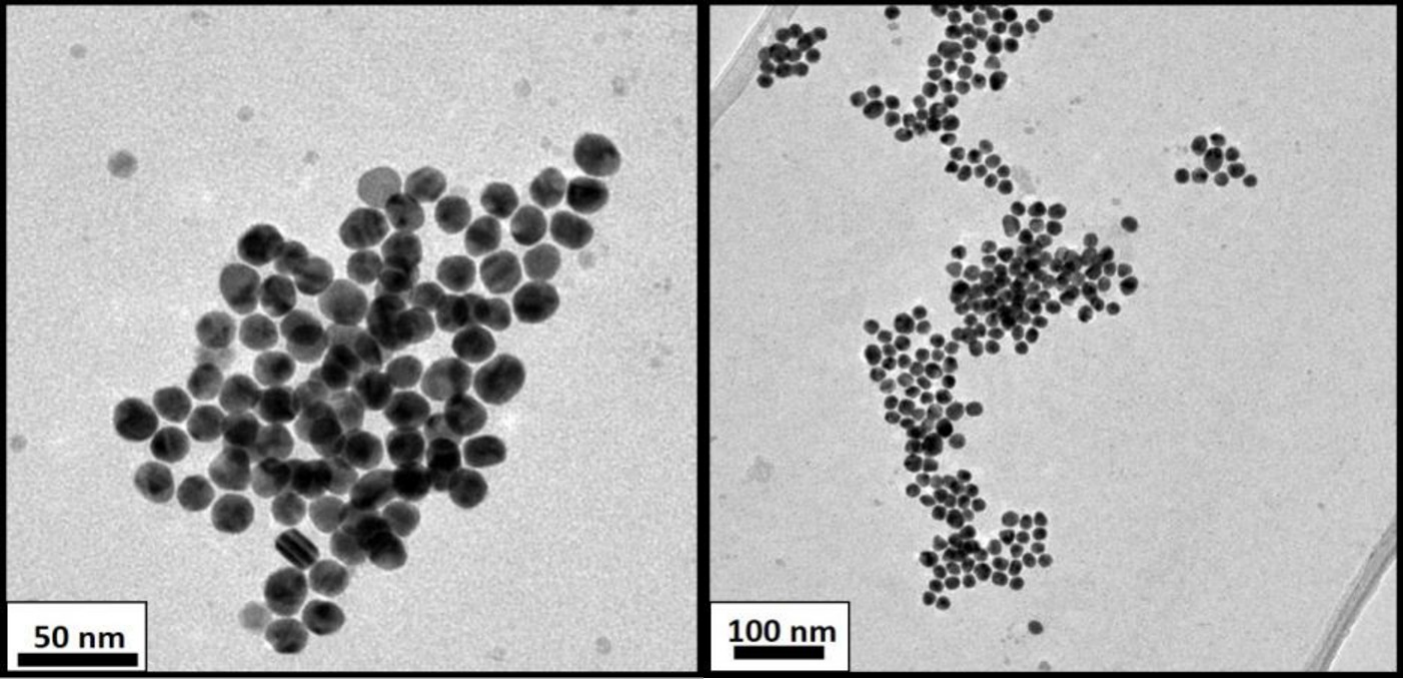
TEM micrographs of AuNP after synthesis with citrate.

HAuCl_4_ (25 mM) was added to Milli-Q water, using citrate as a stabilizing agent. After 1 min of stirring, sodium citrate (5 mM) was added to the chloroauric solution. One minute later, ice-cold freshly prepared NaBH_4_ (0.1 M) solution was added to the mixture. The colloidal solution was stirred for 5 min, and then the nanoparticles were characterized by TEM as shown in Fig 1.

The conjugation of 11-Mercaptoundecanoic acid (MUA) (Sigma Aldrich) was performed based on the procedure described by Comenge *et al*. [8,11]. The MUA solution required heat with stirring and heated sonication for 15 min to fully dissolve the solid, and basified with NaOH 2M per ml of solution.

### Concentration of MUA-AuNP

HCl buffer was added to the MUA-AuNP. The destabilized solution was then centrifuged for 40 min at 12,500 rpm. The supernatant was removed, and the nanoparticle pellet was resuspended in 28 mM, pH = 8.0 borax (Sigma Aldrich)/HCl buffer.

The AuNP solution was further diluted 1:2 in physiological media to reach a final concentration of 2.5 mM.

### Cisplatin conjugation

The conjugation of cisPt to MUA-AuNPs was performed based on the procedure developed by Comenge *et al*. [8], by using borate buffer. The solution was stirred gently for 25 min.

**Fig 2:**
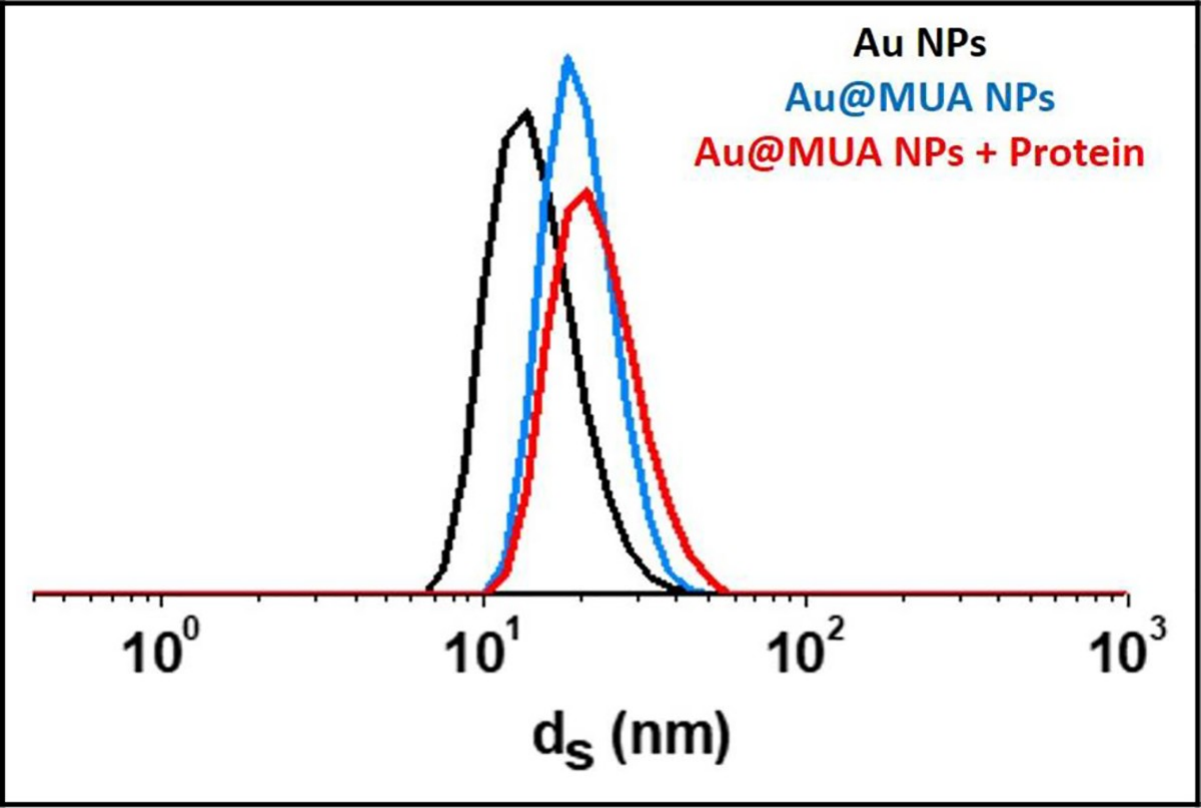
AuNP particle size (nm) by DLS measurements (in % volume) depending on the gold bindings. AuNP (black), AuNP-MUA (blue), AuNP-MUA diluted in cellular medium rich in proteins (red).

The final Dynamic Light Scattering (DLS) characterization showed a particle diameter of around 14 nm, ligand included (see Fig 2). TEM images are shown in Fig 1 to study the size dispersion and compare the size with DLS measures.

### Flow cytometry analyses to assess cellular damage after treatments

Flow cytometry analyses were performed 24 h after drug incubations. To obtain the correct percentage of each cell population, it was essential to combine the floating and trypsinized cells and measure them together before performing the test using the Annexin-V-FLUOS staining kit (Roche). Samples were analysed by using a FACS Calibur (Becton Dickinson). Flowing free software (Turku Centre for Biotechnology, University of Turku, Finland) was used for the final data analysis.

### Resazurin cell metabolic evaluation assay (QBlue test)

To perform the resazurin or QBlue cell viability test (Qblue Cell Viability Assay kits, BioChain, USA), the remaining living cells were trypsinized and counted immediately after each treatment. Subsequently, ten thousand cells were seeded per well in a 96-well plate and incubated at 37°C in order to check the cell recovery of living cells at different time intervals. In this study, QBlue assays were performed at days two (D2), five (D5) and six (D6) after cisPt-based treatments.

Finally, fluorescence measurements were carried out with a Wallac Fluorometry plate reader (excitation wavelength at 550 nm and emission at 615 nm) at room temperature.

### Transmission Electron Microscopy (TEM)

Living cells were fixed 24 h after treatments with 2% (w/v) paraformaldehyde (Sigma-Aldrich) and 3% (v/v) glutaraldehyde (EM grade, Merck, Darmstadt, Germany) in 0.1 M phosphate buffer (Sigma-Aldrich), following the protocol described elsewhere [14]. Briefly, the samples were post-fixed with OsO_4_ 1%, washed several times with distilled H_2_O, and dehydrated in acetone, embedded in Eponate resin at 60°C for 48 h, and sectioned with an ultramicrotome. Finally, ultrathin sections placed in copper grids were contrasted with uranyl acetate and Reynolds lead citrate solutions, and examined using a Jeol 1400 (Jeol LTD, Japan) TEM equipped with a CCD GATAN ES1000w Erlangshen camera.

### Cryo-synchrotron xr microscopy (Cryo-SXT)

Sample preparation was performed in the cell culture lab at ALBA, where A431NS human cSCC cells were seeded on gold quantifoil (R2/2) holey carbon grids (Au-G200F1) and incubated for 24 h at 37°C and 5% CO_2_. Then, all supernatants were removed and replaced by the different chemical conditions (i.e. cisPt free and cisPt with adjuvants) described before.

Samples were vitrified by plunge freezing in a Leica EM-CPC. The frozen grids were imaged using a LINKAM CMS196 stage in a Zeiss AxioScope fluorescence microscope. The frozen grids were transferred to Mistral (ALBA-Light Source) [15,16] beamline at ALBA synchrotron under cryogenic conditions. The photon energy was set within the water window (520 eV) in order to take advantage of the high natural absorption contrast of the biological material to acquire X-ray tomography data sets under the conditions described previously [17]. The datasets were acquired using a zone plate objective with an outermost zone width of Δr_n_ = 25 nm. The effective pixel size in the images was 9.8 nm. The image stacks were pre-processed to normalize and correct the intensity distribution delivered to the sample by the capillary condenser lens.

Alignment of the tilted series was effected with IMOD [18] and the final reconstructions were made using the iterative SIRT reconstruction option in TOMO3D [19]. To enhance the signal to noise ratio, TOMOEED was used [20]. Visualization and manual segmentation (i.e. segmentation of the surface boundaries identifying different organelles to color-code them) of the final volumes was carried out with IMOD and Chimera [21]. The 3D-resolution estimation was done using the same approach as previously described [17].

### Statistical analysis of data

All experiments were performed at least three times. Final data are evaluated using a standard one-factor analysis of variance (ANOVA) test in order to determine how the cell viability varied depending on each treatment. Probability (*p*) values obtained from Bonferroni’s test greater than 0.05 were used to indicate non-significant differences (ns), whereas asterisks denoted the following cutoff differences between the groups: *p < 0.05, **p < 0.001, ***p < 0.0001.

The results for control cells (cells without any treatment) obtained from flow cytometry were considered as having 100% survival, and 0% of both death and early apoptotic indexes. The results for all the cellular populations (analyzed by flow cytometry and QBlue test) were normalized to the control group and expressed as a mean percentage ± standard deviation (SD) in the Figures.

## Results

**Fig 3:**
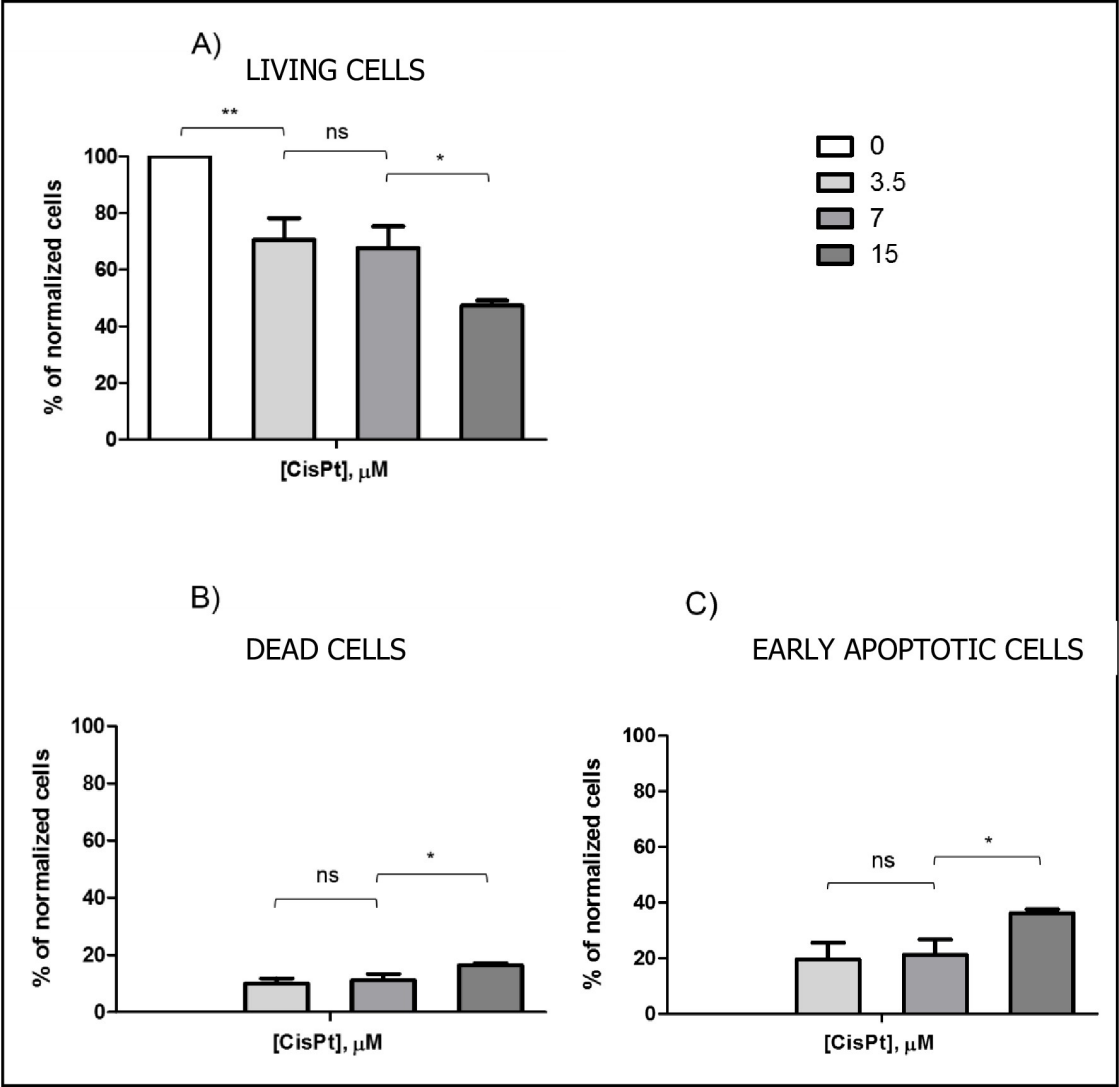
Normalized histograms obtained by flow cytometry 24 h after cisPt incubation at three concentrations: 3.5, 7 and 15 μM. The plots correspond to (A) living cells, (B) completely dead cells, and (C) cells undergoing early stage apoptosis. Results are expressed as mean percentages ± SD.

Cells were treated at three cisPt concentrations 3.5, 7 and 15 μM in order to determine the effect of cisPt at very low concentrations on cSCC cells. Flow cytometry results 24 h after cisPt incubation are shown in Fig 3.

CisPt caused a significant decrease in the percentage of living cells, especially at 15 μM (47.3% ± 1.8) (see Fig 3A). In all cases, apoptosis was the main cell death pathway (Fig 3B and 3C), with double the number of cells undergoing early apoptosis (**3.5** μ**M:** 10.0% ± 1.8, **7** μ**M:** 11.1% ± 2.3, **15** μ**M:** 16.4% ± 0.7) with respect to those dying by late apoptosis or necrosis (**3.5** μ**M:** 19.5% ± 6.0, **7** μ**M:** 21.2% ± 5.5, **15** μ**M:** 36.3% ± 1.3). These results indicated that the concentration of cisPt used was not high enough–even at 15 μM- to induce acute cell damage and hence, necrotic cell death. This would have some biological advantages of the treatment, as it implies lower toxicity to the surrounding tissue, and a lack of microcalcifications in that area [22,23].

**Fig 4:**
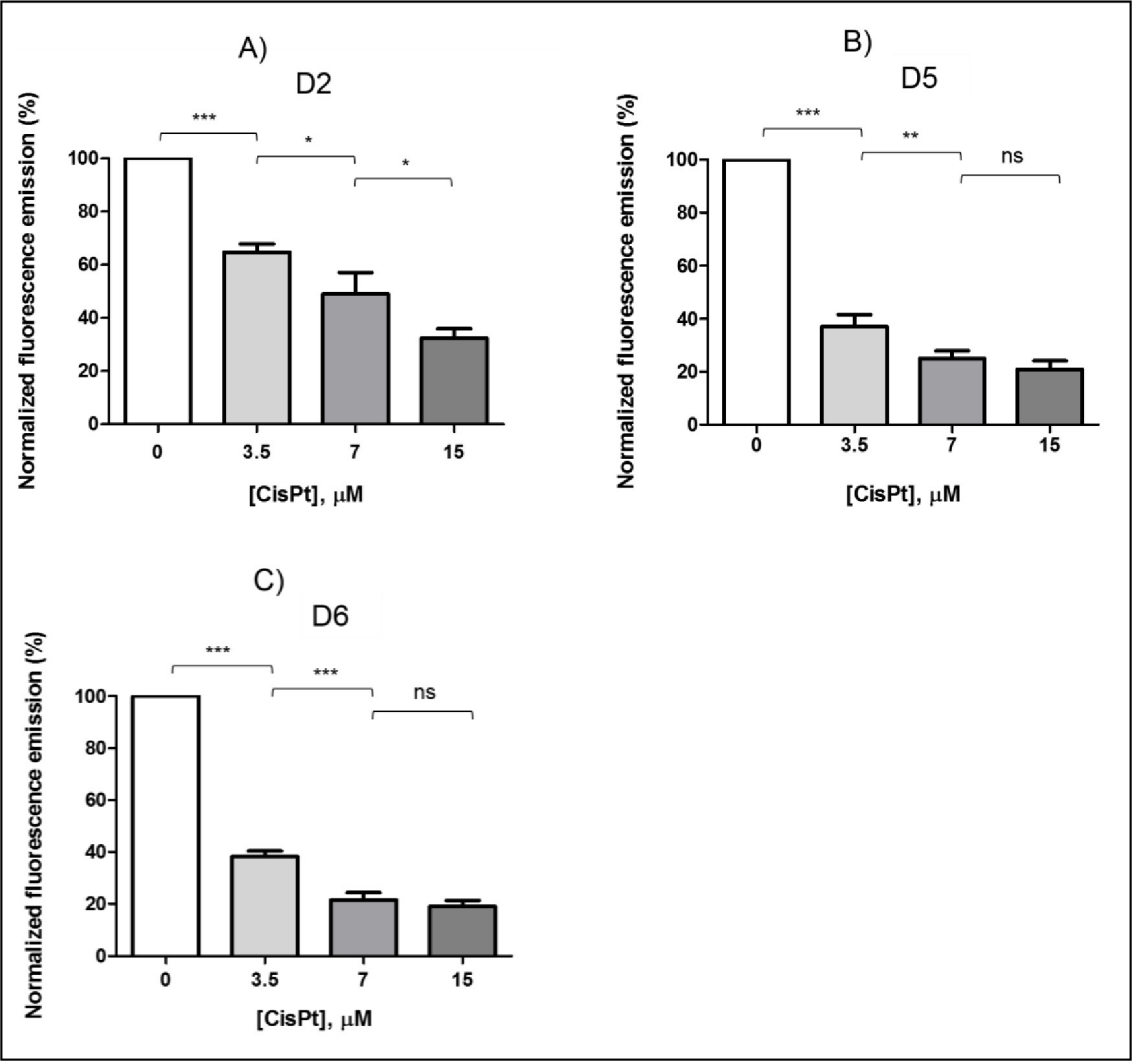
Normalized histograms obtained by QBlue test after cisPt incubation at several concentrations: 3.5, 7 and 15 μM. The plots correspond to QBlue tests performed at (A) day 2 (D2), (B) day 5 (D5), and (C) day 6 (D6) after treatment. Note that cisPt 7 and 15 μM were the most aggressive treatments, with a lower cell recovery ability up to day 6.

According to the results obtained by flow cytometry, cisPt 3.5 μM apparently caused the same effect on cSCC cells as cisPt 7 μM. However, analyzing the ability of living cells to recover over time, differences between both treatments could be detected. These results are shown in Fig 4A–4C.

In particular, the first analysis performed 72 h (A in Fig 4) after cisPt-based treatments showed a lower metabolic cell activity–and thus lower cell recovery- with cisPt 7 μM (48.9% ± 4.36) than with cisPt 3.5 μM (64.8% ± 2.36). The difference in metabolic cell activity between these concentrations was increased on D5 (Fig 4B), and even more on D6 (Fig 4C), when the effects of cisPt 7 μM on cell metabolism were found to be the same that those detected with cisPt 15 μM.

Therefore, the working concentration for cisPt could be assessed at 3.5 μM since this may decrease side effects on healthy tissues. Moreover, at this dose, cisPt caused ~30% of cell death, of which ~20% was via apoptosis. The metabolic activity of the remaining living cells, i.e. their recovery ability, decreased with time up to 38.2% ± 1.52 at D6, but it was not as low as these found for 7 and 15 μM.

**Fig 5:**
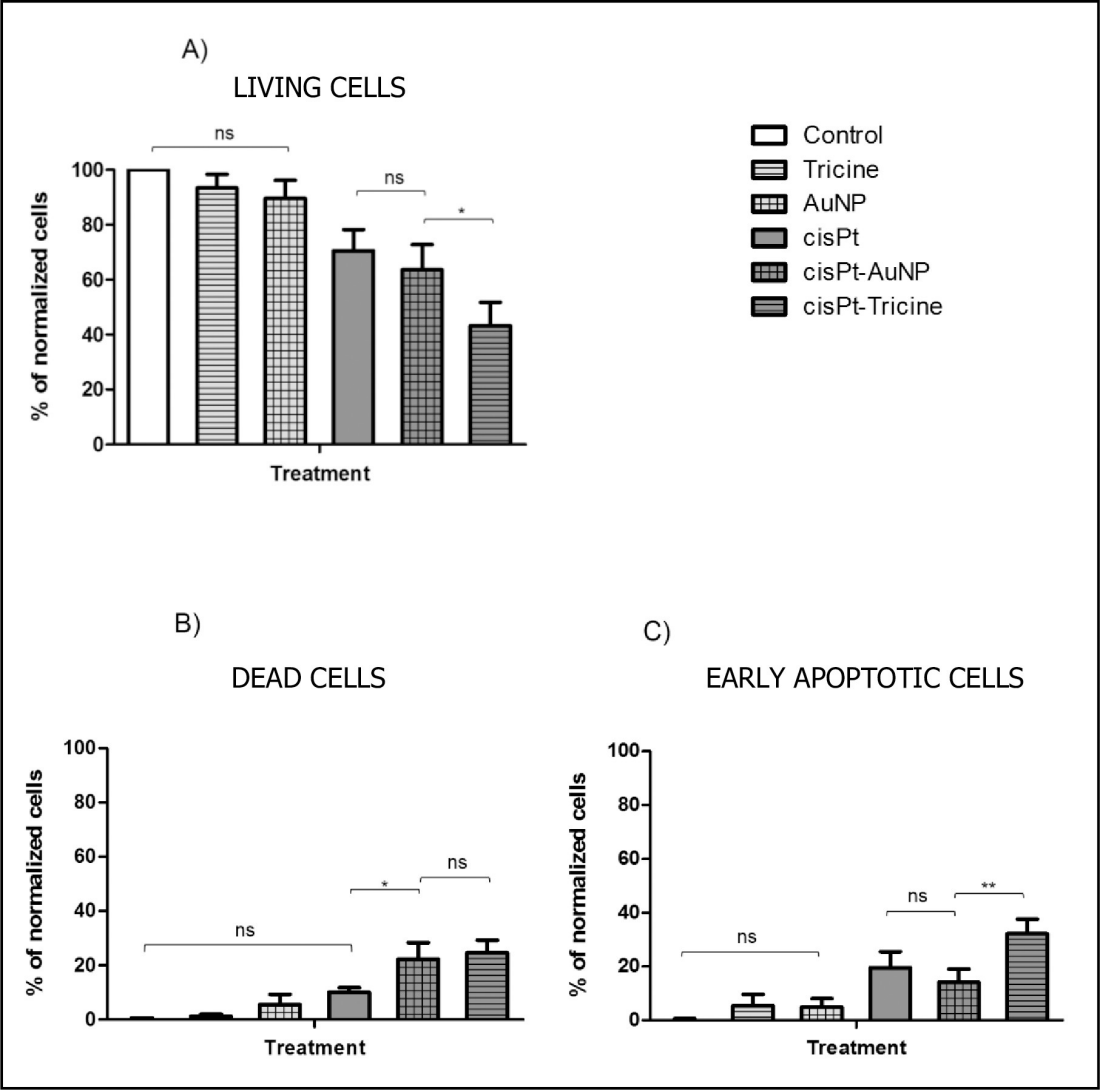
Normalized histograms obtained by flow cytometry 24 h after cisPt 3.5 μM alone, associated with AuNP or mixed with tricine. The plots correspond to (A) living cells, (B) completely dead cells, and (C) cells undergoing early stage apoptosis. Results are expressed as mean percentages ± SD.

The use of adjuvant agents such as AuNP and tricine would enhance the effectiveness of cisPt at this low concentration (i.e. 3.5 μM), while keeping healthy tissues undamaged. The results on cSCC by means of flow cytometry are shown in Fig 5.

Incubation of tricine 50 mM alone did not present any toxic effect on cells as shown in Fig 5A. However, tricine mixed with cisPt 3.5 μM led to the most significant decrease in the living cell population (43.2% ± 8.5) with respect to the control group. Therefore, treatment based on tricine and cisPt was the most effective, leading to a preferential apoptotic death cell (32.2% ± 5.4).

Similar results were observed for AuNP-MUA (diameter, 14 nm), as the nanoparticles alone did not seem to be toxic *in vitro*, but in combination with cisPt, the proportion of damaged cells increased significantly (see Fig 5A–5C). In fact, AuNP were expected to be toxic only if their size was less than ~10 nm, which corresponds to the pore size of nuclear membrane, meaning they could enter the nucleus and bond covalently with DNA [24].

The interesting feature of these data is the comparison of living cell percentages between cisPt-AuNP (63.5% ± 9.3) and free cisPt 3.5 μM (70.5% ± 7.7): no differences were detected as can be seen in Fig 5C. This could be due to a lack of efficient covalent bonding between AuNP-MUA and cisPt molecules. However, the death pathway for each treatment was observed to be different as cells treated with free cisPt undergo apoptosis (Fig 5C) as expected in accordance with the results shown in Fig 3C, whereas cisPt-AuNP caused a higher proportion of late-apoptotic and necrotic dead cells (both referred to as dead cells in Fig 5B). This finding would suggest that some molecules of cisPt were probably electrostatically linked to AuNP-MUA, i.e. through an unexpected weak bond, and they could enter the cell more easily with respect free cisPt, causing more damage to it, thus leading to an increase in necrotic cell death.

Cellular images obtained by TEM and cryo-SXT confirmed the results obtained by means of flow cytometry for each chemotherapy treatment.

**Fig 6:**
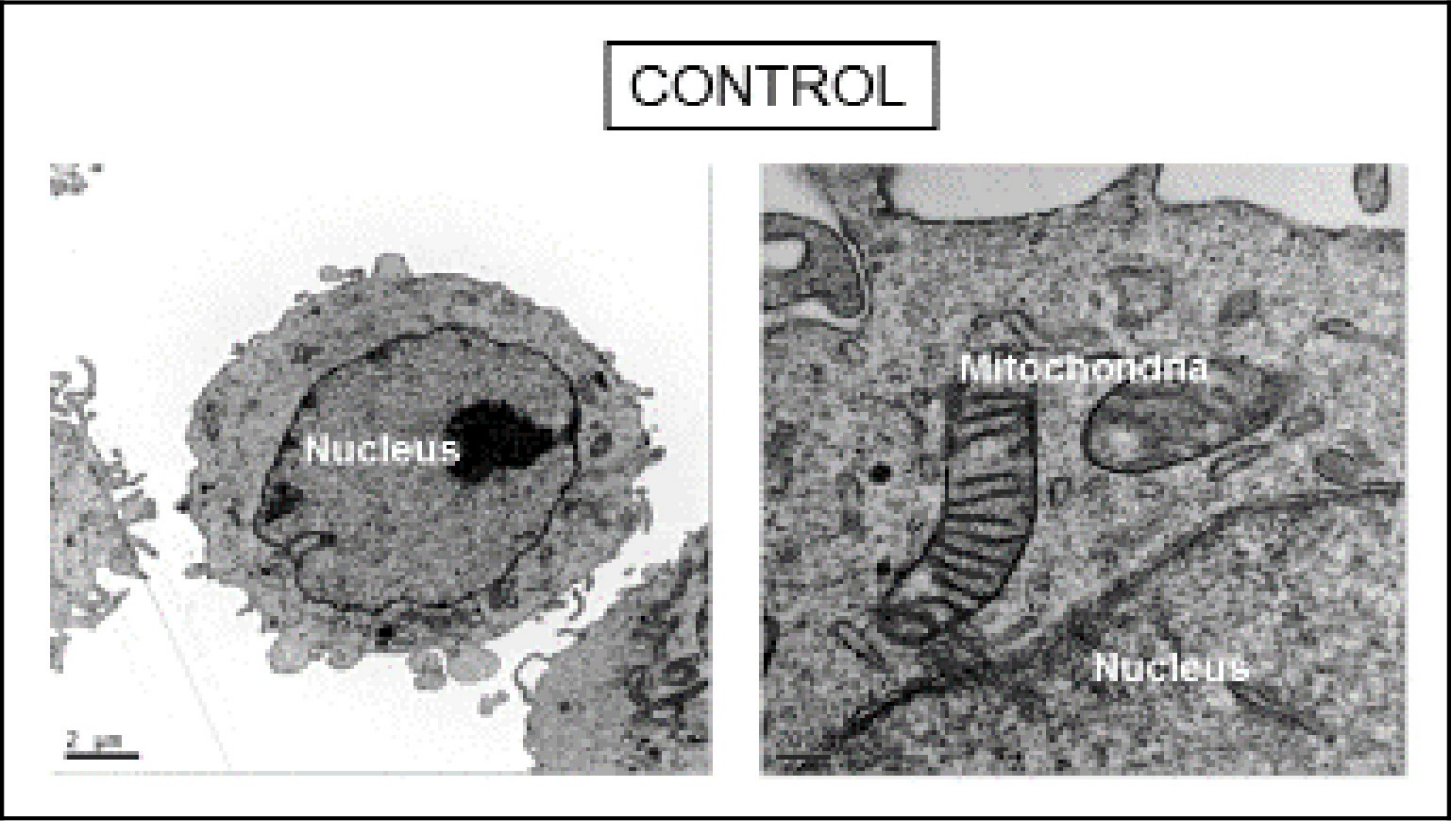
TEM micrographs of control cells. All membranes and organelles, including mitochondria, were intact.

**Fig 7:**
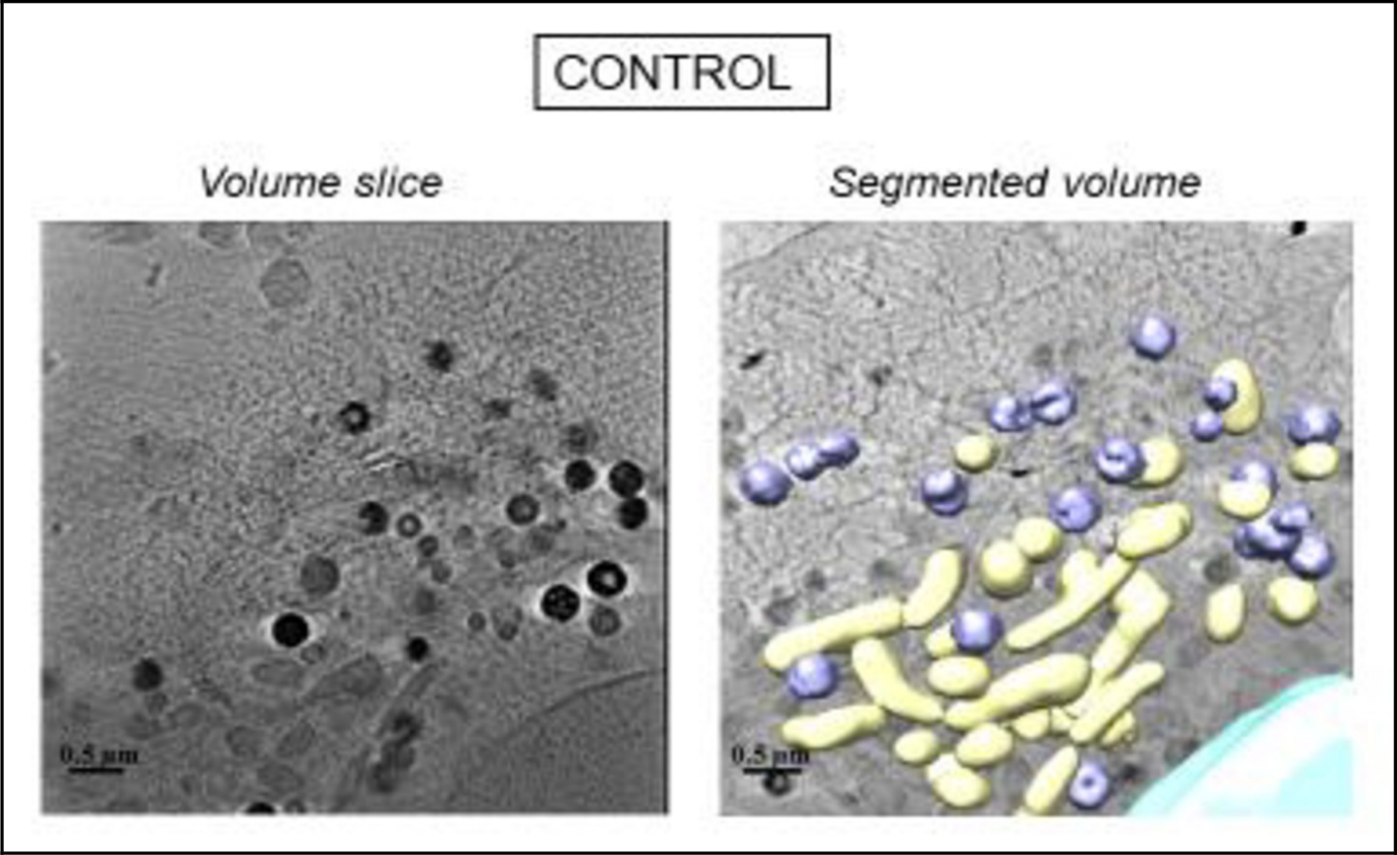
Three-dimensional reconstruction of whole-cell volumes by cryo-SXT tomography of control cell (manual surface segmentation). A volume slice of the tomogram is shown. Color-coded manual segmentation of the surface boundaries identifying different organelles is seen in the control cell: mitochondria (yellow), the nucleus (blue), endosomes and/or lysosomes (purple).

Untreated cells, referred to “Control” in Figs 6 and 7, showed normal morphological features with both microscopy techniques. Granular structures corresponding to chromatin fibers could be identified in the nucleus of control cells both by TEM (Fig 6) and cryo-SXT (Fig 7), segmented in blue. Another notable feature of control cells was the presence of structures with a high degree of contrast in the cytoplasm due to their high carbon content which may correspond to lipid droplets, endosome compartments of the endocytic pathway, and/or lysosomes (in purple in Fig 7). Cryo-SXT of the cells revealed the presence of other recognizable organelles such as mitochondria which were clearly recognizable by both cryo-STX and TEM.

**Fig 8:**
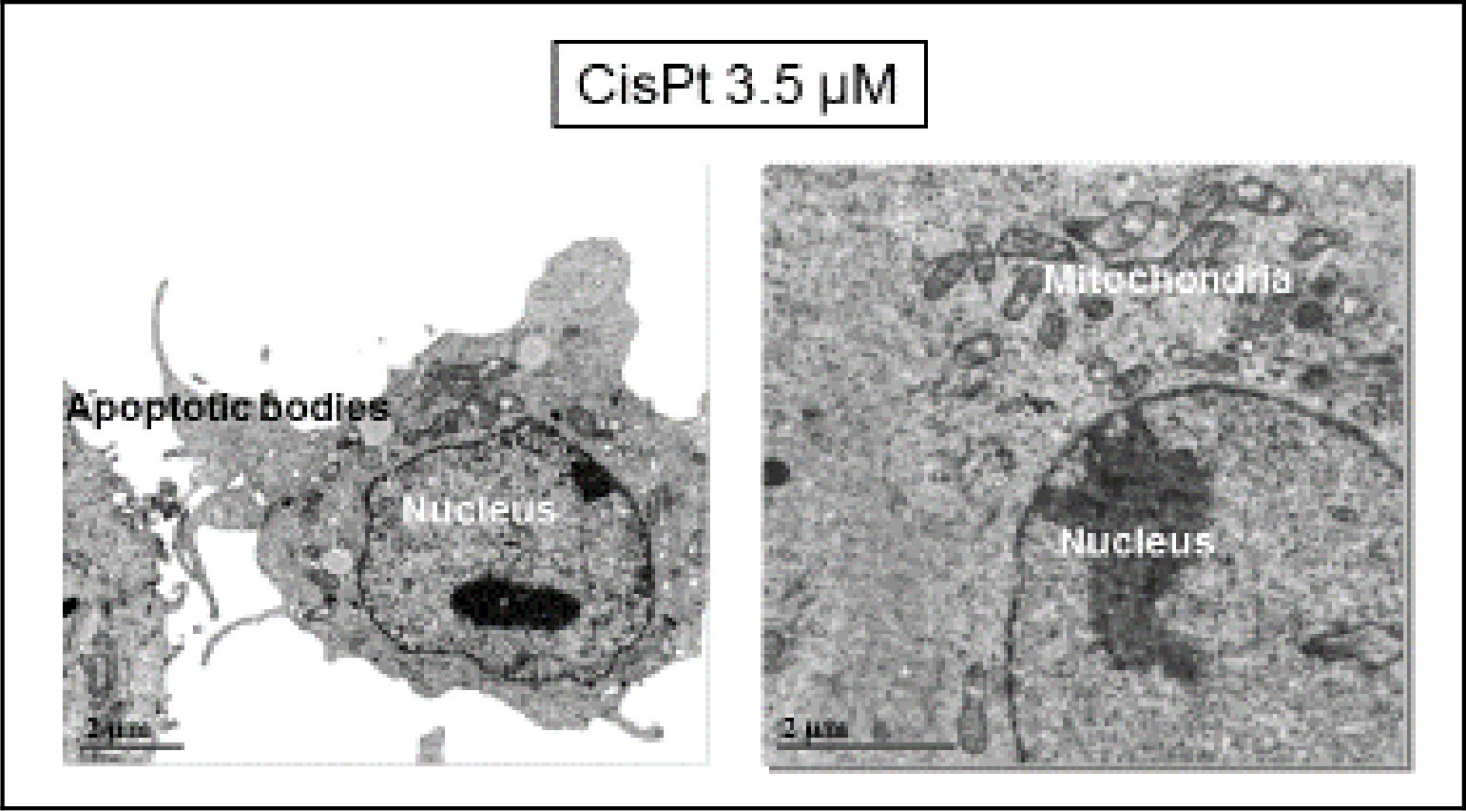
TEM micrographs of cisPt-treated cells. Some apoptotic bodies appeared on the cell surface, whereas mitochondria as well as the other organelles seemed not to suffer damage.

**Fig 9:**
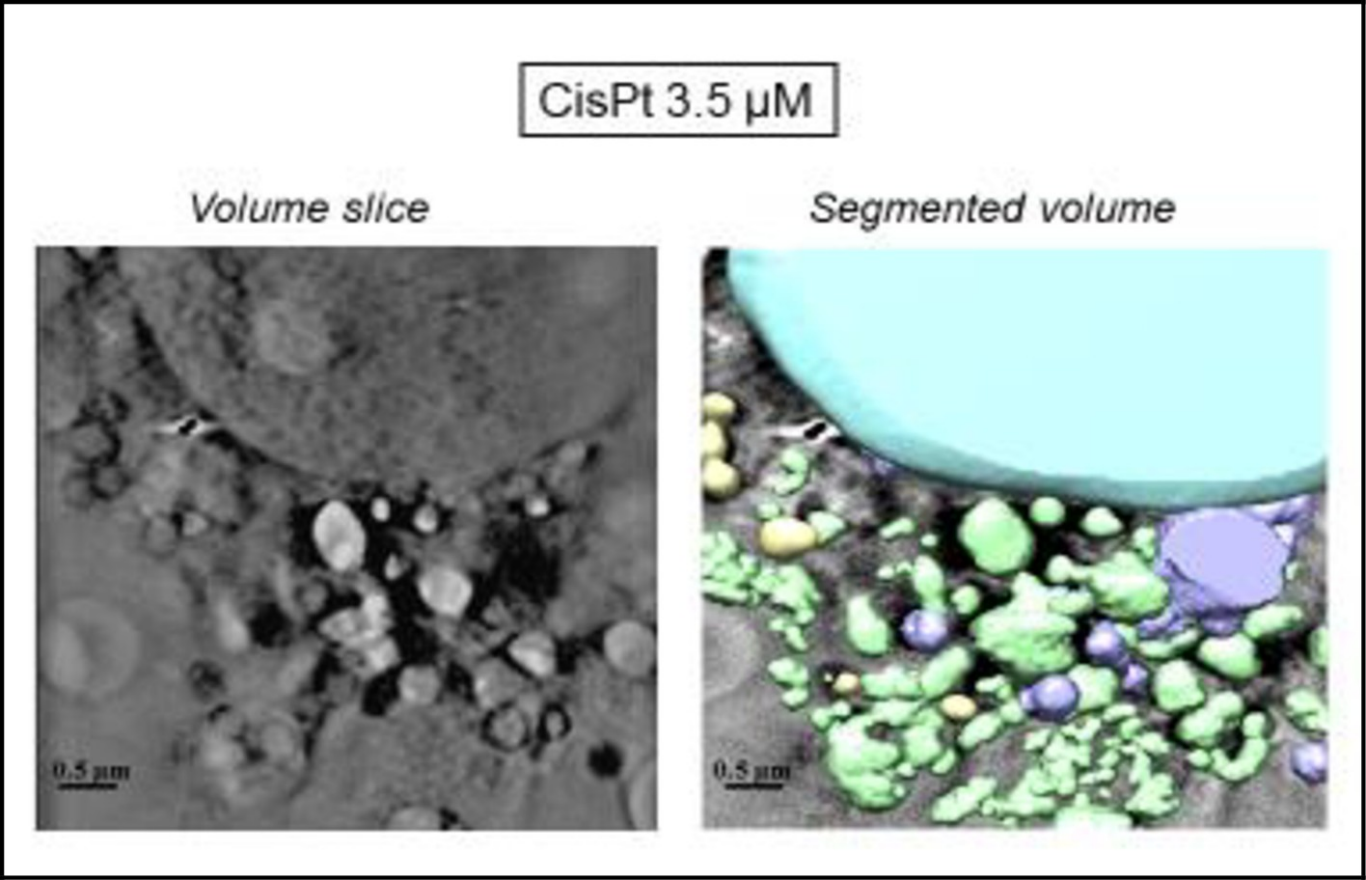
Three-dimensional reconstruction of whole-cell volumes by cryo-SXT tomography of cisPt-treated cell. A volume slice of the tomogram is shown. Color-coded manual segmentation of the surface boundaries identifying different organelles is seen in the cisPt-treated cell: mitochondria (yellow), the nucleus (blue), endosomes and/or lysosomes (purple) and vacuoles (green).

CSCC cells incubated with cisPt (see Figs 8 and 9) displayed cell-surface morphological changes typical of apoptosis such as cell membrane blebbing (Fig 8), and vacuoles (Fig 9) on it. Moreover, segmentation through the low absorbance at 520 eV (Fig 9) demonstrated that mitochondria were now more condensed than in the control cells.

In order to further explore the effects of tricine and AuNP when combined with cisPt, pretreated cells were analyzed in detail by cryo-SXT at ALBA light source. This technique allowed the tomography of individual cells, leading to a 3D image of treated samples, and thus, a higher resolution in comparison with TEM.

**Fig 10:**
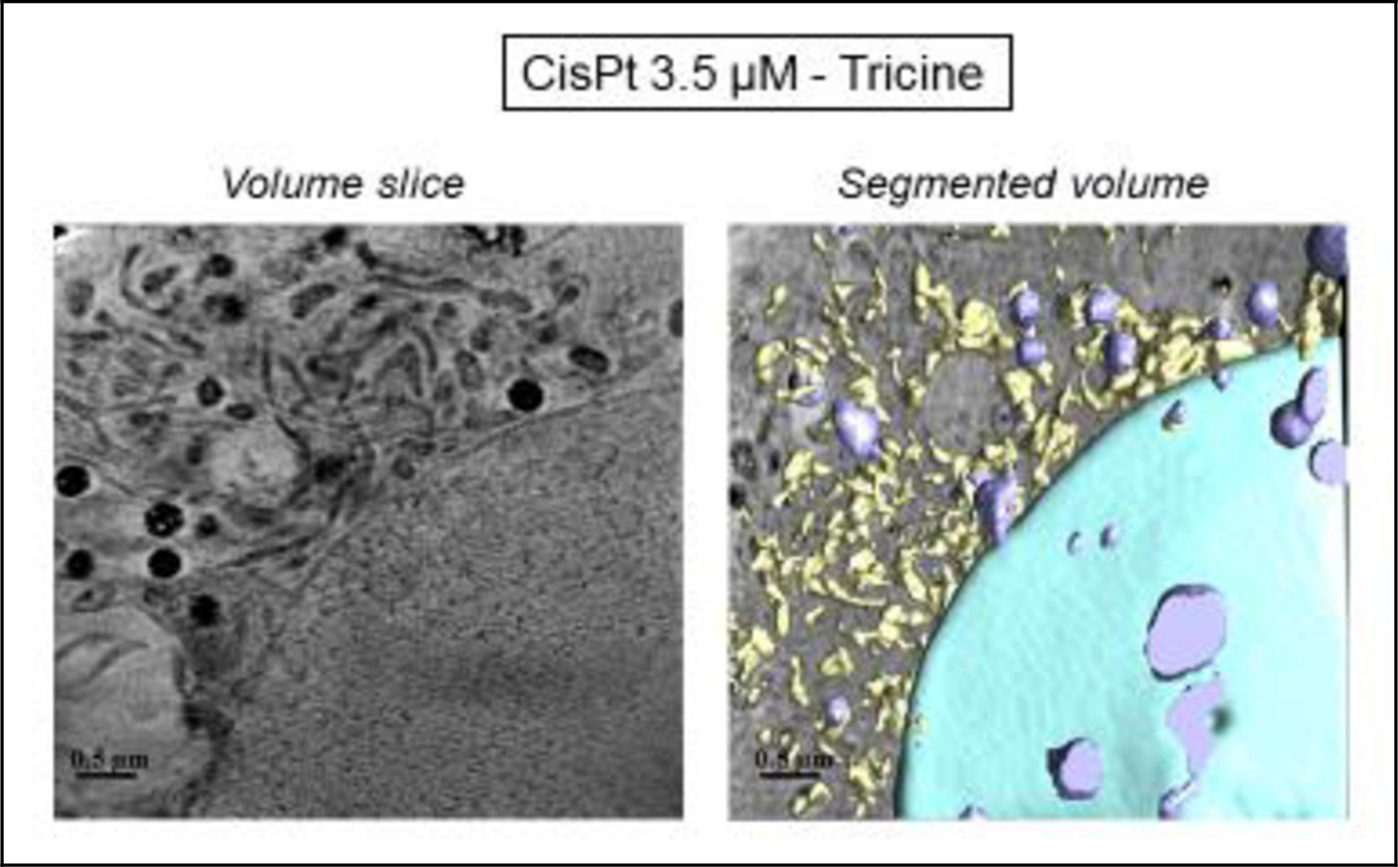
Three-dimensional reconstruction of whole-cell volumes by cryo-SXT tomography of cells treated with cisPt and tricine. A volume slice of the tomogram is shown. Color-coded manual segmentation of the surface boundaries identifying different organelles was seen in the cisPt-tricine-treated cell: mitochondria (yellow), the nucleus (blue), endosomes and/or lysosomes (purple).

With regard to microscopic results, cisPt 3.5 μM (Fig 8) and cisPt-tricine-treated cells (Fig 10) presented similar features in their plasmatic membranes, with morphological changes on it consistent with apoptosis.

The cytoplasm of the cells treated with cisPt and tricine (Fig 10) showed nuclear chromatin condensation, prominent mitochondria, and some cytoplasmic vacuolation. The increased mitochondrial proportion confirmed that cSCC cells could undergo an energy-consuming death process after the treatment. Thus, cisPt-incubated cells, either free or with tricine, exhibited morphological changes typical of the early phase of apoptosis. Results from cryo-SXT were in accordance with the data obtained by flow cytometry for each chemotherapy treatment: the combination of tricine and cisPt emerged as the most effective strategy.

**Fig 11:**
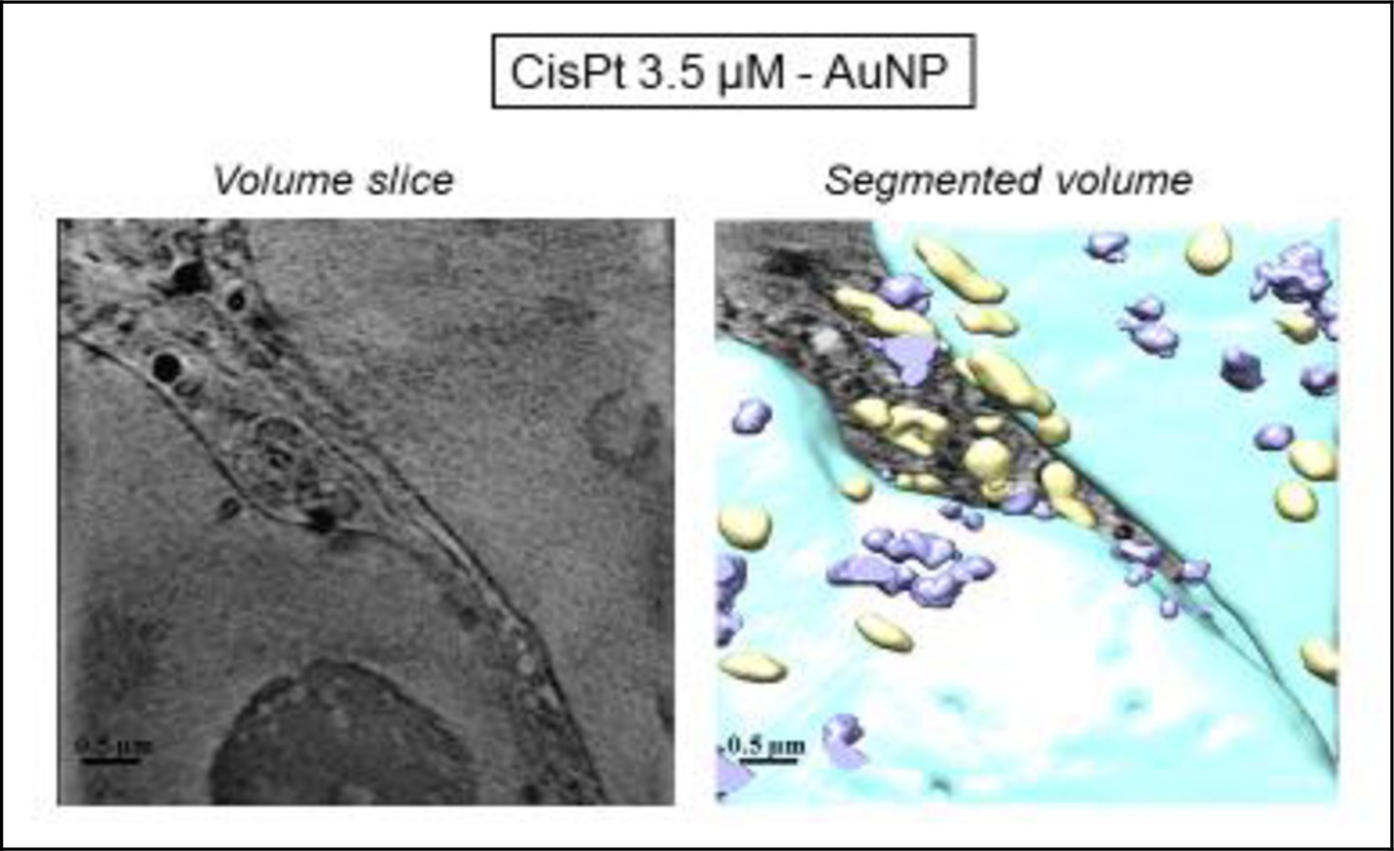
Three-dimensional reconstruction of whole-cell volumes by cryo-SXT tomography of cells treated with cisPt and AuNP. A volume slice of the tomogram is shown. Color-coded manual segmentation of the surface boundaries identifying different organelles is seen in cisPt-AuNP-treated cells: mitochondria (yellow), the two nuclei (blue), endosomes and/or lysosomes (purple).

**Fig 12:**
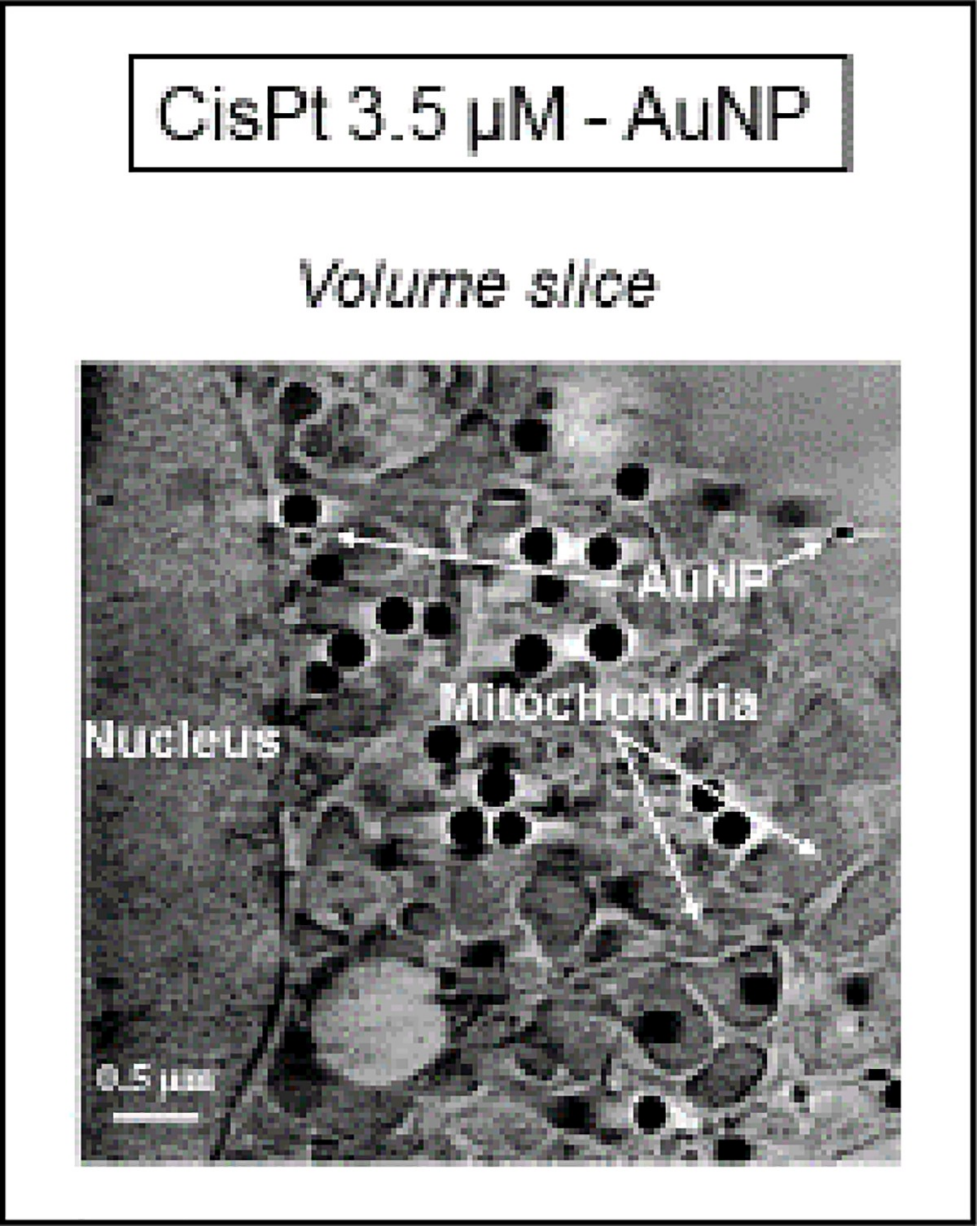
Volume slice of cryo-SXT tomography of a cell treated with cisPt and AuNP. Different organelles are detected within the cytoplasm of cisPt-AuNP-treated cell, as well as those treated with AuNP. Note that AuNP appeared close to the nucleus, i.e. in the perinuclear area.

According to the cryo-SXT results (Fig 11), mitochondria (yellow) as well as the other of organelles seemed not to be damaged. AuNP were visible in the cytoplasm, but they did not enter the nucleus (Fig 12). Hence, cisPt-AuNP did not seem qualitatively as effective as cisPt-tricine, since the cell seemed to be almost undamaged in Fig 11. CSCC cells treated with cisPt associated with AuNP showed normal euchromatin in the nucleus, with an intact nuclear membrane (Figs 11 and 12). Prominent mitochondria, slight vacuolation and the presence of vesicles were some features that were observed in these samples. Results from flow cytometry were also in line with these findings. In addition, nanoparticles were distributed within the cytoplasm, especially in the perinuclear area (Fig 12).

The initial hypothesis that AuNP, due to their size, did not enter the nucleus, and thus, did not cause cell toxicity by itself, was also confirmed by the X-ray synchrotron images. Nevertheless, AuNP-MUA would enhance the cisPt entry into the cell through an unexpected weak bond between them, increasing necrotic cell death.

## Discussion

The high potential of cisPt as a chemotherapy agent against cSCC with respect to 5-fluorouracil has already been described [7]. Some other studies also suggested platinum compounds as an alternative treatment to inoperable cSCC [25]. Going one step further, the results obtained in this work showed that cisPt 3.5 μM diluted in tricine 50 mM was the most effective strategy to significantly decrease (up to ~40%) cSCC cell survival with only one application. These results were clearly better in terms of effectiveness than those obtained previously in treatments with cisPt 15 μM, and without any adjuvant agent [7].

Tricine was chosen to be combined with cisPt as it is widely used as a buffer to reach the required physiological conditions [9], and it is also used in nuclear medicine to achieve stabilization of the radionuclides [26]. Moreover, tricine is also a compound of interest due to its ability to form unidentate, bidentate, and tridentate complexes with metal ions [27]. This flexibility is largely dependent on the reagent concentration, as well as the pH of the solution.

The hypothesis for the enhancement of cisPt effectiveness in the presence of tricine might be copper chelation. Tricine is expected to be prone to bind intracellularly with Cu^2+^ ions rather that Pt^2+^ atoms. This is due to the higher affinity of the several active chelator centers of tricine to copper rather than other metallic atoms [27,28]. This would, therefore, facilitate entry into the cell through the Cu-transporter proteins (hCtr1) of more positive-charged ions such as platinum (i.e. cisPt). This view is supported by Zheng *et al*. 2012 [29], who observed the same enhancement of cisPt effectiveness on cancer cells when it was combined with copper-chelating agents.

Results of treatments using AuNP-MUA as an adjuvant suggest, that cisPt might need to be activated prior to each application, in order to bind covalently to AuNP, as suggested by Comenge *et al*. 2010 [11]. This could explain the fact that no differences were found in the effectiveness of free cisPt and cisPt-AuNP, regardless of the technique used for analysis.

Despite the lack of covalent bonding between AuNP-MUA and cisPt molecules, some molecules of cisPt might be electrostatically linked to AuNP-MUA, allowing their entry into the cell more easily, and causing more damage to it, showing a slight increase of necrotic cell death (Fig 5B). This fact differed from tricine, which in combination with cisPt led to a preferential apoptotic death mode, as well as to a better adjuvant for cisPt treatments. Nevertheless, the quantification of the cisPt concentration within cells as a function of the adjuvant agent could be estimated from the cellular extracts. Thus, the validation of higher uptake of cisPt associated with tricine could be assessed.

Cryo-SXT allows tomography of individual cells, leading to a 3D image of treated samples, and thus, not only was a higher resolution feasible in comparison with TEM, but also a volumetric cell analysis after each treatment. This technique allowed the 3D volume investigation, and hence a deeper evaluation of AuNP as an adjuvant could be performed. Results indicated that cisPt-AuNP did not seem qualitatively as effective as cisPt-tricine, as shown by flow cytometry data. In addition, nanoparticles were distributed within the cytoplasm, especially in the perinuclear area. The lowest resolution of TEM did not allow to obtain images of internalized NP, in contrast with cryo-SXT.

Moreover, tomographies obtained by cryo-SXT allowed a color-coded manual segmentation of the surface boundaries in order to better identify the different organelles. Cryo-SXT was the only image-based technique that allowed 3D investigation on a nanometric scale.

## Conclusions

Results suggest that there was no covalent bonding between AuNP-MUA and cisPt molecules under the experimental conditions described, although some molecules of cisPt might be electrostatically linked to AuNP-MUA, allowing easier entry into the cell, and causing more damage to it, resulting in a slight increase in necrotic cell death. Conversely, treatment based on cisPt 3.5 μM combined with tricine 50 mM enhanced drug effectiveness, increasing cell death, especially via apoptosis, and was therefore a better adjuvant for cisPt treatment. Despite this positive result, the mechanism underlying the effectiveness of the combination of cisPt and tricine is still uncertain, and further research to elucidate that point is needed.
